# Efficacy and safety of sivelestat sodium hydrate adjunct to remifentanil for analgesia in patients under mechanical ventilation (SIDING trial): protocol of a multicenter, randomized, double-blinded, placebo-controlled trial

**DOI:** 10.3389/fmed.2026.1907083

**Published:** 2026-09-30

**Authors:** Zongwen Zhang, Peng Wang, Bin Yao, Wenjian Mao, Weibang Pan, Xiaoxia Zhu, Shifeng Li, Jun Wang, Yue Ai, Jiangtao Yin, Binhua Xue, Jun Liu, Yiyuan Pan, Jinlong Wang, Min Shao, Lening Ren, Yingying Hu, Lin Gao, Lu Ke, Zhihui Tong, Yuxiu Liu, Xiangcheng Zhang, Weiqin Li

**Affiliations:** 1 Center of Severe Acute Pancreatitis (CSAP), Department of Critical Care Medicine, Jinling Hospital, Affiliated Hospital of Medical School, Nanjing University, Nanjing, Jiangsu, China; 2 Center of Severe Acute Pancreatitis (CSAP), Department of Critical Care Medicine, Jinling Clinical Medical College, Nanjing Medical University, Nanjing, Jiangsu, China; 3 Department of Intensive Care Unit, The Affiliated Huai'an No.1 People’s Hospital of Nanjing Medical University, Huai'an, Jiangsu, China; 4 Department of Critical Care Medicine, The First Affiliated Hospital of Soochow University, Suzhou, Jiangsu, China; 5 Department of Critical Care Medicine, The Affiliated Hospital of Jiangsu University, Zhenjiang, Jiangsu, China; 6 Department of Critical Care Medicine and Emergency, The Affiliated Suzhou Hospital of Nanjing Medical University, Suzhou Municipal Hospital, Gusu School of Nanjing Medical University, Suzhou Clinical Medical Center of Critical Care Medicine, Suzhou, Jiangsu, China; 7 Department of Critical Care Medicine, The First Affiliated Hospital of Anhui Medical University, Hefei, Anhui, China; 8 Department of Critical Care Medicine, The First Affiliated Hospital of Henan University of Science and Technology, Luoyang, Henan, China; 9 National Institute of Healthcare Data Science, Nanjing University, Nanjing, Jiangsu, China

**Keywords:** analgesia, opioids, remifentanil, sivelestat sodium, ventilation

## Abstract

**Introduction:**

Inadequate analgesia is associated with worse outcomes in patients under mechanical ventilation. Current guidelines recommend opioids as the first-line analgesics. However, prolonged use of opioids is associated with numerous adverse effects. Preclinical studies have shown that sivelestat sodium hydrate attenuates pain-related behaviours in rodent models; however, its analgesic and opioid-sparing effects in humans remain unknown. This study aims to investigate whether sivelestat reduces remifentanil requirements while maintaining adequate analgesia in mechanically ventilated patients.

**Methods and analysis:**

This multicenter, randomized, double-blinded, placebo-controlled trial is conducted in seven ICUs across China. 136 patients under mechanical ventilation will be randomly assigned to the placebo and sivelestat groups at a 1:1 ratio. The study drug will be administered at an initial rate of 0.2 mg/kg/h, as an adjunct to a standard remifentanil-based analgesic regimen. Remifentanil will be used as the protocolized background analgesic, with rescue analgesics permitted when predefined criteria are met. The primary outcome is mean hourly remifentanil consumption per kilogram during the intervention period.

**Ethics and dissemination:**

This study has been approved by the ethics committee of The Affiliated Huai'an No.1 People's Hospital of Nanjing Medical University (KY-2024-093-01) and all participating sites. Results will be disseminated through peer-reviewed journals and scientific conferences.

**Study Protocol Registration:**

https://www.chictr.org.cn, identifier ChiCTR2400086632.

## Introduction

Pain management is a fundamental component of care for patients receiving mechanical ventilation in the intensive care unit (ICU). Inadequate analgesia can lead to increased patient-ventilator asynchrony, agitation, and unplanned extubation, associated with prolonged duration of mechanical ventilation, higher complication rates, and increased mortality (1, 2). Opioids are considered first-line analgesics in this setting (3, 4). However, high cumulative doses of opioids are associated with numerous adverse effects, including delirium, respiratory depression, delayed gastrointestinal motility, and immunosuppression (5–7). Current guidelines and previous studies recommend the adjunctive use of non-opioid analgesics with different mechanisms of action as part of a multimodal analgesia strategy, aimed at reducing opioid consumption and improving analgesic efficacy (4, 8). Multimodal analgesia is also associated with more rapid extubation (9).

Inflammation plays a key role in the modulation of pain. Notably, neutrophil elastase (NE), a serine protease released by activated neutrophils during systemic inflammation, can directly promote nociception via protease-activated receptor 2 (PAR-2)/transient receptor potential vanilloid 4 (TRPV4) sensitization in sensory neurons (10, 11). This effect is direct and not mediated by opioid degradation. Sivelestat sodium hydrate, a selective NE inhibitor, is approved in China for the treatment of acute lung injury associated with systemic inflammatory response syndrome. Preclinical studies have reported that sivelestat attenuates pain-related behaviours in rodent models of peripheral nerve injury, osteoarthritis, painful diabetic neuropathy, and cancer pain (11–13). These findings provide a biological rationale for investigating sivelestat as an adjunctive therapy but do not establish a direct analgesic effect in humans.

While clinical studies on sivelestat to date have primarily focused on pulmonary outcomes, its potential impact on analgesic requirements remains unknown (14–16). Therefore, this trial is designed to evaluate the opioid-sparing effect and safety of sivelestat in mechanically ventilated patients. We hypothesize that the addition of sivelestat to a remifentanil-based analgesic regimen will significantly reduce remifentanil consumption while maintaining adequate analgesia and sedation.

## Methods and analysis

### Study design and setting

This is a multicenter, randomized, double-blinded, placebo-controlled trial. The design followed the Standard Protocol Items: Recommendations for Interventional Trials guideline (17). This trial has been approved by the ethics committee of The Affiliated Huai'an No.1 People's Hospital of Nanjing Medical University (KY-2024-093-01) and all participating sites, and has been registered at the Chinese Clinical Trials Registry (ChiCTR2400086632). Flowchart of enrolment, interventions and assessments are provided in Figure 1 and Table 1.

**Figure 1:**
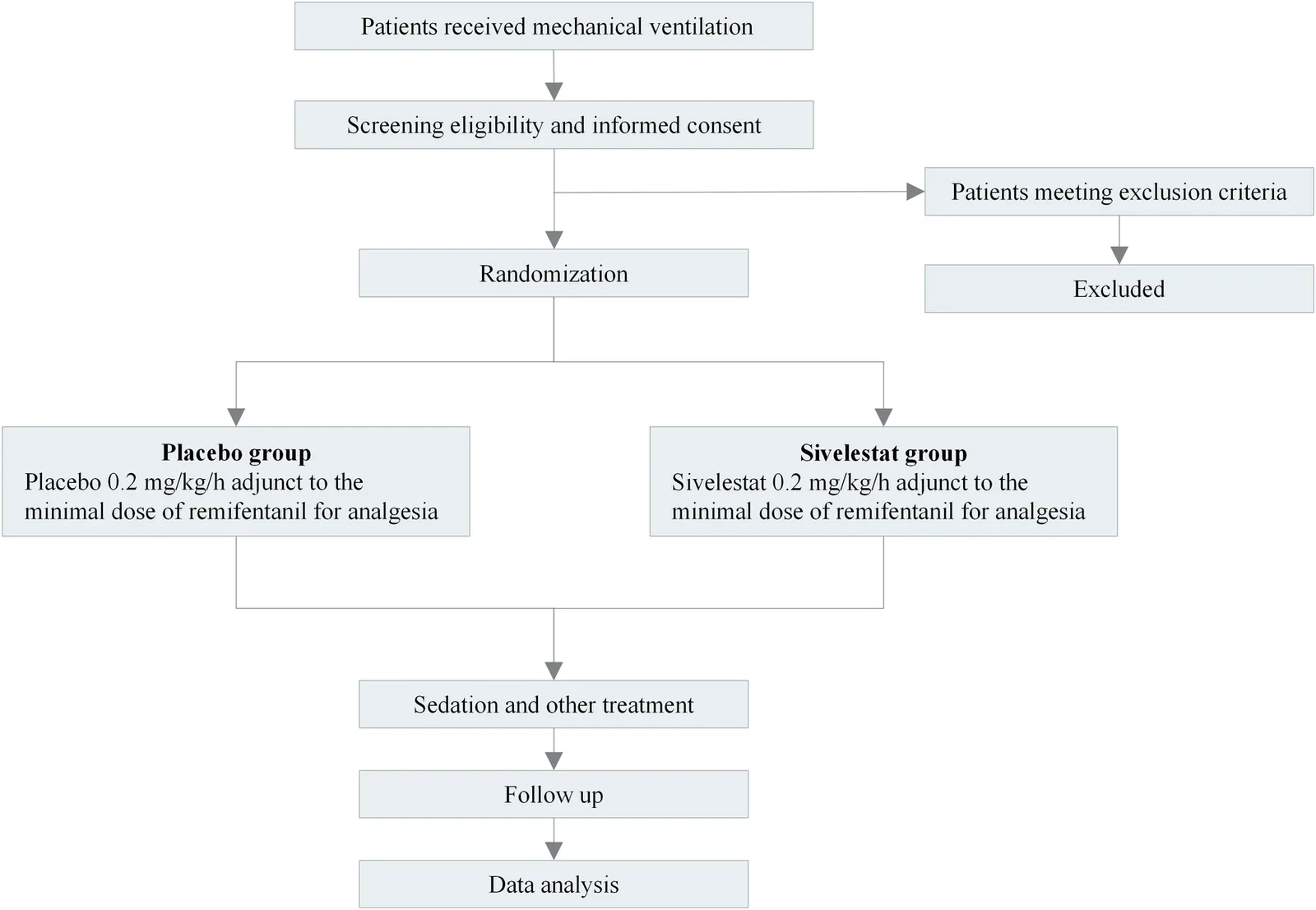
Flowchart.

**Table 1:** Study schedule.

Time point	Day −1	Day 0	Day 1	Day 2	Day 3	Day 4	Day 5	Day 6	Day 7	Day.	Day 28	Day 90	Day 180	Discharge
Enrolment														
Eligibility screening	×													
Informed consent	×													
Allocation		×												
Intervention		×	×	×	×	×								
Assessment														
Baseline variables	×													
Diagnosis	×													
Comorbidity	×													
Mechanical ventilation duration before randomization		×												
Cumulative dose of remifentanil, midazolam, propofol, dexmedetomidine before randomization		×												
SOFA and APACHE II score		×			×				×					
Enteral nutrition and target rate					×				×					
GIDS, enteral nutritional tolerance score and intra-abdominal pressure		×			×				×					
RASS and CPOT（per 4 h）		×	×	×	×	×								
Cumulative dose of study drug, remifentanil, midazolam, and propofol		×	×	×	×	×								
Cumulative duration of study drug, remifentanil, midazolam, and propofol		×	×	×	×	×								
CAM-ICU and psychotropic drugs used for delirium			×	×	×	×			×		×			×
Requiring frequent suctioning			×	×	×	×								
Uncontrolled agitation			×	×	×	×								
Biochemical measurements			×		×				×					
Adverse events			×	×	×	×	×	×	×	×	×			×
Mechanical ventilation free days and vasopressor free days			×	×	×	×	×	×	×	×	×			×
Length of ICU stay														×
Length of stay														×
Mortality rate											×	×	×	

SOFA, Sequential Organ Failure Assessment; APACHE, Acute Physiology and Chronic Health Evaluation; GIDS, Gastrointestinal Dysfunction Score; RASS, Richmond Agitation-Sedation Scale; CPOT, Critical Care Pain Observation Tool; CAM-ICU, Confusion Assessment Method for the Intensive Care Unit; ICU, Intensive Care Unit.

This multicenter trial is currently being conducted in the ICUs of seven tertiary hospitals in China, including The Affiliated Huai'an No.1 People's Hospital of Nanjing Medical University, Jinling Hospital, The First Affiliated Hospital of Soochow University, The Affiliated Hospital of Jiangsu University, The Affiliated Suzhou Hospital of Nanjing Medical University, The First Affiliated Hospital of Anhui Medical University, The First Affiliated Hospital of Henan University of Science and Technology.

### Study population

The inclusion and exclusion criteria are as follows:

#### Inclusion criteria

1. Adult patients (aged ≥18 years);
2. Patients who were intubated and mechanically ventilated within 24 h, with an expected duration of ventilation >48 h post-randomization;
3. Non-surgical patients (defined as having undergone no surgery of Grade 2 or above within the past week, according to the “Management Measures for Surgical Grading in Medical Institutions” of the Chinese Ministry of Health);
4. Written informed consent obtained from the patients or next of kin;

#### Exclusion criteria

1. Pregnancy or lactation;
2. Inability to reliably assess Richmond Agitation-Sedation Scale (RASS) and Critical Care Pain Observation Tool (CPOT) due to the medical condition;
3. Contraindications to sivelestat sodium, remifentanil, propofol, midazolam or their excipients;
4. Requiring deep sedation (defined as RASS score ≤−4) and/or continuous infusion of neuromuscular blocking agents;
5. Suspected or confirmed acute primary brain injury, including but not limited to: traumatic brain injury, cerebral infarction, intracranial hemorrhage, spinal cord injury, hypoxic ischemic encephalopathy, hydrocephalus, or cerebral oedema.
6. Severe cardiac pathology, including acute myocardial infarction, cardiogenic shock or severe systolic dysfunction (left ventricular ejection fraction <30%);
7. Estimated glomerular filtration rate (eGFR) < 30 mL/min/1.73 m^2^ (using CKD-EPI formula);
8. End-stage liver disease (Child-Pugh class C);
9. Patients with malignant disease who are undergoing radiotherapy or chemotherapy and require additional analgesia;
10. Requiring surgery or tracheotomy within the next 48 h;
11. History of active drug or alcohol abuse/dependence;
12. Receiving palliative care or with a life expectancy of less than 48 h;
13. History of dementia, major psychiatric disorder, or current requirement for psychotropic medication;
14. Concurrent participation in another interventional clinical trial, or participation in such a trial within the preceding 30 days;
15. No clinical requirement for opioid analgesia, as assessed by the treating clinician.

### Recruitment and informed consent procedure

Written informed consent will be obtained from all patients or their next of kin after they have received and understood the benefits and potential risks of the trial and have had the opportunity to discuss the trial with the investigator. Randomization and study intervention will begin after obtaining written informed consent.

### Randomization, allocation and concealment

Eligible patients are randomized in a 1:1 ratio to either the sivelestat group or the placebo group using permuted block randomization, stratified by study site. A fixed block size of four was used to maintain treatment balance within each study site. The randomization sequence and block structure were generated and retained by an independent statistician and were inaccessible to investigators and clinical staff. Identical, sequentially numbered study-drug kits were dispensed to enrolled participants strictly in numerical order, thereby ensuring allocation concealment.

### Blinding

This study employs a double-blind design to ensure that both the participants and the clinical team members remain blinded to the treatment assignment until the end of the study. Sivelestat and matched placebo were supplied by Shanghai Huilun (Jiangsu) Pharmaceutical. The placebo contained the same excipients without sivelestat, and the two preparations were identical in appearance, packaging, and labeling. After reconstitution, they were also matched for color, clarity, and foaming. Preparation volume, diluent, mixing procedure, and standing time will be standardized.

### Interventions

#### Study drug and analgesic management

Within one hour of randomization, a continuous intravenous infusion of the study drug (sivelestat or matched placebo) will be initiated at an initial rate of 0.2 mg/kg/h, as an adjunct to a standard remifentanil-based analgesic regimen. The remifentanil regimen consists of a loading dose (1.5 µg/kg) followed by a continuous maintenance infusion of no more than 30 µg/kg/h (18). Patients who have already received remifentanil loading and maintenance infusions before randomization will continue at their existing maintenance rate without an additional loading dose. Previous exposure to fentanyl, sufentanil, or other opioids will be converted to morphine-equivalent doses and recorded in the database, after which analgesia will follow the protocolized remifentanil titration algorithm.

#### Titration protocol

The remifentanil infusion will be titrated to maintain a CPOT score ≤ 2 (19). CPOT will be assessed every 30 min after study drug initiation and after each remifentanil adjustment by the site investigator. Based on each assessment, the remifentanil infusion rate will be adjusted according to a predefined algorithm: the dose will be reduced by 10%, if CPOT is ≤2, or increased by 10%, if CPOT is >2. This process aims to identify and maintain the minimum effective remifentanil dose required to achieve the analgesic target. Once CPOT ≤2 is maintained at two consecutive assessments with no further infusion change, assessments or re-titration will revert to every 4 h. For patients with a CPOT score ≥6, the investigator will first check for potentially reversible causes of pain. A slow intravenous bolus of remifentanil 0.5 μg/kg will then be given over 60 s, together with a 25%–50% increase in the continuous infusion rate. CPOT and vital signs will be reassessed within 5 min under continuous cardiorespiratory monitoring. If CPOT remains ≥6, the investigator will reassess the cause of pain, the clinical response, and the patient's safety before determining further management, including the use of additional analgesic agents when appropriate. Patients with a CPOT score ≤5, the titration protocol will be resumed (20). Remifentanil may be reduced to a minimum infusion rate of 0 μg/kg/h. If CPOT ≤ 2 is maintained after discontinuation of remifentanil, the infusion rate of the study drug may be reduced in increments of 0.05 mg/kg/h while continuing to monitor the CPOT score. The analgesic dosing algorithm for the study is shown in Figure 2. If remifentanil infusion rate reaches the predefined upper limit of 30 µg/kg/h and the analgesia remains inadequate, additional analgesic agents are permitted as part of a multimodal analgesia strategy. The use of all analgesics will be recorded for subsequent analysis. If hypotension, bradycardia, respiratory depression, or another potentially opioid-related adverse event occurs, alternative causes will first be assessed and treated. Remifentanil will be reduced or discontinued when the event is considered opioid-related.

**Figure 2:**
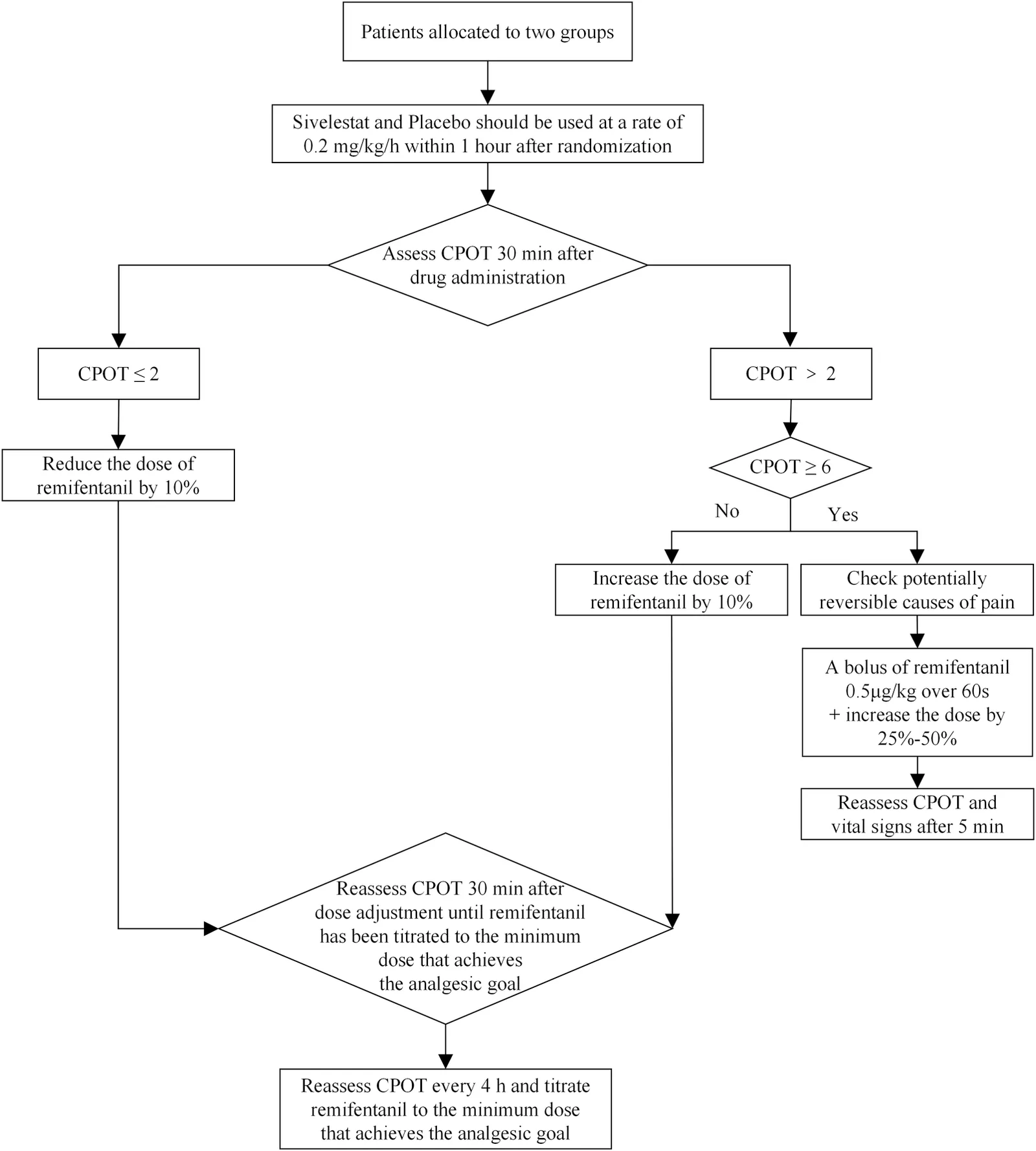
Analgesics dosing algorithm.

#### Management of sedation

Sedation management will follow the international guideline recommendations and will be identical for both treatment groups. The protocol-defined light-sedation range is RASS −2 to +1, consistent with the PADIS guideline (4, 21). RASS +1 represents the acceptable upper boundary rather than the preferred sedation state and prompts assessment of reversible causes. Propofol is the primary sedative agent, with midazolam as the permitted alternative. A standardized sedation protocol, developed in accordance with guideline recommendations and the eCASH (early Comfort using Analgesia, minimal Sedatives and maximal Humane care) principle, is implemented for all patients (Figure 3) (22). When RASS is <−2, reversible causes of impaired consciousness will be evaluated, and the sedative medication will be reduced or temporarily withheld as appropriate.

**Figure 3:**
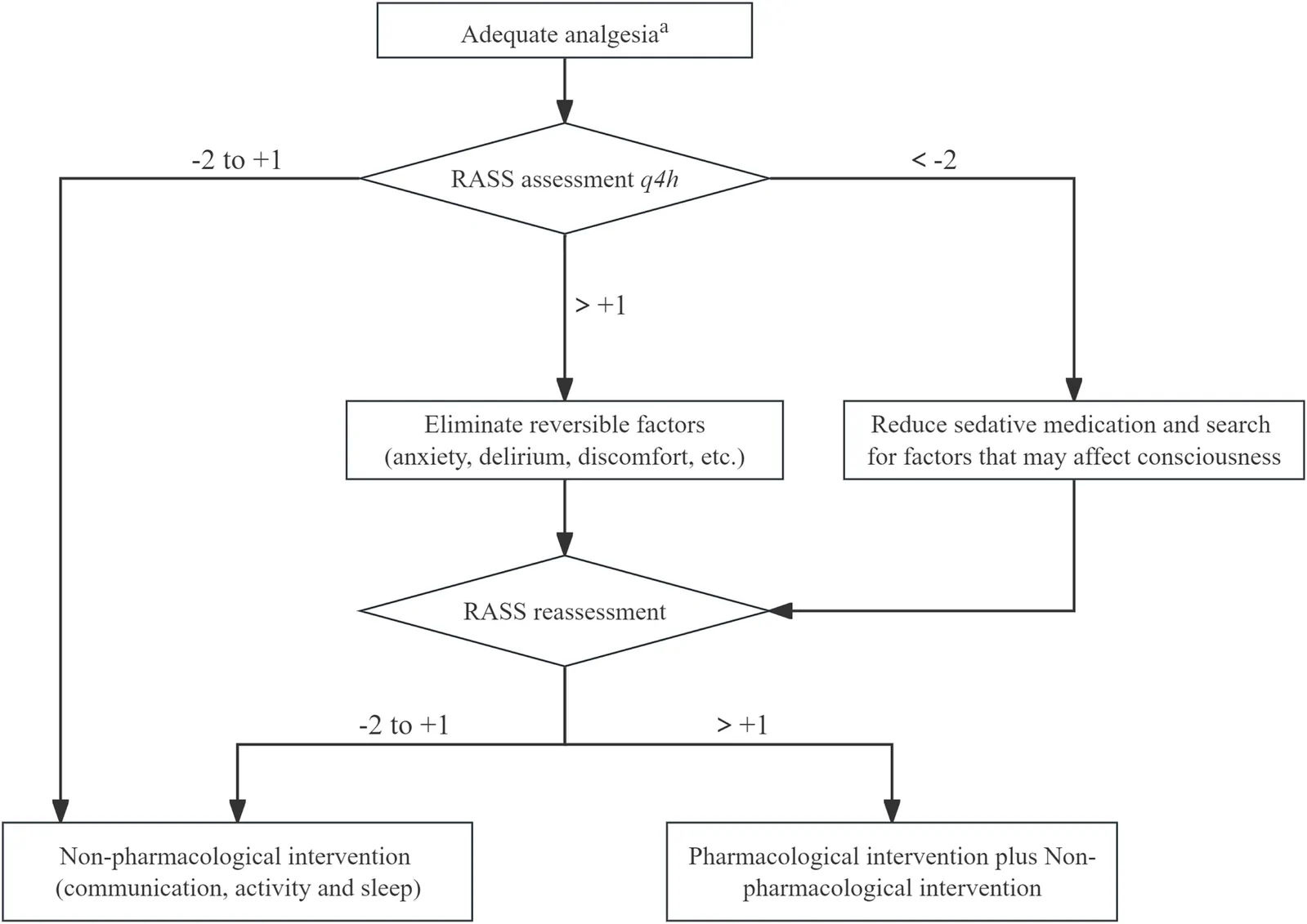
Sedation algorithm. a: Adequate analgesia means CPOT ≤ 2.

#### Other therapies

Management of delirium, immobilization and sleep disturbance will follow the recommended guideline (4). Nutritional therapy for all patients will be provided in accordance with the “Nutrition Support Process for Critically Ill Patients” and other relevant clinical guidelines (23, 24). The treatment of the primary disease and any comorbidities will be guided by applicable guidelines and determined by the treating medical team. Other symptomatic and supportive treatments will be provided at the discretion of the clinical team as required by the patient's condition. Mechanical ventilation will be individualized; liberation from mechanical ventilation will follow the guideline recommendations (25).

#### Duration of the intervention

The intervention will stop when the following events occur (whichever occurs first): (1) 96 h after randomization; (2) the patient or family members withdraw informed consent; (3) the patient dies; (4) analgesics not required due to extubation or other medical reasons, as determined by the treatment team; (5) the patient requires deep sedation or neuromuscular blocking agents; (6) patient requires surgery or tracheotomy; (7) unable to accurately assess CPOT and RASS scores due to changes in disease status; (8) serious adverse event (SAE) occurs (see definition in the Adverse events section).

#### Unblinding and withdrawal of participants

In the event of a serious medical emergency where knowledge of the treatment allocation is considered essential for immediate clinical management, a formal request for unblinding may be submitted. This request requires the agreement of two independent senior clinicians not involved in the trial. The unblinding will be performed in a controlled manner by the independent pharmacy team. The date, time, reason, and individuals involved in the unblinding decision will be fully documented.

Any participant whose treatment allocation is revealed through the emergency unblinding procedure will be discontinued from further administration of the study drug. If a participant withdraws consent or requires discontinuation of the study intervention for any other reason prior to completing the scheduled 96-hour intervention period, efforts will be made to collect available follow-up data, subject to the participant's consent.

#### Follow-up

All patients will be followed for 180 days after randomization. All-cause mortality will be assessed at 28, 90, and 180 days.

### Data collection and management

A web-based electronic data capture (EDC) system has been established for this study. Access is restricted to the principal investigator and authorized coinvestigators at each participating site via individual accounts. All data entered into the EDC system must be traceable to the original source documents (e.g., medical records, case report forms). Investigators are responsible for the accuracy and compliance of data entry at their site. The EDC system also incorporates automated range checks and logical validation rules to minimize data entry errors at the point of entry. If discrepancies, errors, or omissions are identified, a formal data query will be issued to the site. Designated site personnel must resolve all queries by providing clarification or making corrections in the EDC system.

### Outcome measures

#### Primary outcome

Mean hourly remifentanil consumption per kilogram during the intervention period, which is defined as the time from randomization to the end of the intervention. The primary estimand compares sivelestat with placebo, both added to the same remifentanil-based analgesic protocol in all randomized participants. The variable is mean hourly remifentanil consumption per kilogram during the protocol-eligible intervention period, and the population-level summary is the adjusted between-group mean difference. A while-on-treatment strategy is used for intercurrent events terminating protocol-guided analgesia, including extubation, cessation of analgesic requirement, death, deep sedation or neuromuscular blockade, surgery or tracheostomy, inability to assess CPOT/RASS, and SAEs.

#### Secondary outcomes

1. mean hourly consumption and total duration of use for sedatives per kilogram during the intervention period;
2. Target rates for the CPOT and RASS, defined as the proportion of valid scheduled assessments meeting CPOT ≤2 or RASS −2 to +1, respectively, during the intervention period;
3. Proportion of patients requiring frequent endotracheal suctioning (defined as ≥1 episode per 2-hour period) during the intervention period;
4. Incidence of uncontrolled agitation during the intervention period (defined as events of self-extubation, removal of tubes/lines, or aggressive behaviours);
5. Sequential Organ Failure Assessment (SOFA) scores and exploratory assessments of Acute Physiology and Chronic Health Evaluation II (APACHE II) scores on day 3 and day 7 after randomization;
6. Confusion Assessment Method for the Intensive Care Unit (CAM-ICU) and psychotropic drugs used for delirium;
7. Volume of enteral nutrition delivered and the target-reaching rate of nutrition (based on prescribed caloric targets) on day 3 and day 7 after randomization;
8. Gastrointestinal Dysfunction Score (GIDS), enteral nutrition tolerance score, and intra-abdominal pressure on day 3 and day 7 after randomization;
9. PaO_2_/FiO_2_ ratios on day 3 and day 7 after randomization;
10. Biochemical measurements on day 1, day 3 and day 7 after randomization, including white blood cell count, C-reactive protein, alanine aminotransferase, aspartate aminotransferase, total bilirubin, serum creatinine, and platelet count;
11. Incidence of adverse events (AEs) and SAEs;
12. Key clinical outcomes within 28 days after randomization: ventilator-free days, vasopressor-free days, length of ICU stay, length of hospital stay;
13. All-cause mortality at days 28, 90, and 180.

### Adverse events

Investigators and the clinical care team will actively monitor all participants for any AEs and SAEs throughout the trial. An AE is defined as any untoward medical occurrence in a patient administered a medicinal product, which does not necessarily have a causal relationship with this intervention. A SAE is defined as any untoward medical occurrence that results in death, is life-threatening, requires inpatient hospitalization or prolongation of existing hospitalization, results in persistent or significant disability/incapacity, or other unpredictable serious medical events. The causality of all AEs to the study drug will be assessed by the investigator and classified as certain, probable, possible, unlikely, or unrelated. All AEs will be managed appropriately according to clinical practice and recorded in detail in the source documents and the electronic case report form (eCRF). All SAEs will be reported immediately to the principal investigator. The principal investigator is responsible for the timely reporting of all SAEs to the Institutional Review Board in accordance with regulatory and ethical guidelines.

Given that sivelestat is an approved drug with an established safety profile, the intervention is limited to 96 h, the sample size is modest, and no interim analysis is planned, an independent Data Monitoring Committee has not been established. The ethics committee at the coordinating center, while not a formal Data Monitoring Committee, provides relevant oversight, including safety report review. An independent medical safety monitor, who is not involved in trial conduct or data analysis, will review safety data every three months and after any potentially intervention-related SAE. Liver function, coagulation parameters, platelet count, bleeding, and hypersensitivity reactions will be monitored during the intervention. Suspected unexpected serious adverse reactions will prompt discontinuation of the study drug, expedited reporting, and emergency unblinding when necessary. The trial will be temporarily suspended following any potentially related fatal or life-threatening reaction, two similar unexpected serious reactions, or a clinically important imbalance in related SAEs, pending review by the safety monitor and ethics committee.

### Sample size calculation

The sample size was calculated based on the analgesic requirement for remifentanil. A previous study reported a mean remifentanil dose of 0.046 ± 0.020 µg/kg/min for early analgesia in intubated patients (18), equivalent to 2.76 ± 1.20 µg/kg/h, the unit used for our primary outcome. Based on prior experience at Jinling Hospital, the hourly consumption of remifentanil in mechanically ventilated patients may be reduced by approximately 25% when sivelestat is co-administered at a dose of 0.2 mg/kg/h. Assuming a more conservative 22% reduction in hourly remifentanil consumption with sivelestat treatment, a two-group *t*-test with a two-sided significance level (α) of 0.05, a power (1 − β) of 80%, assumed group means of 2.76 and 2.16 μg/kg/h, and a common standard deviation (σ = 1.20) was applied. The calculation, performed using PASS 2022 software, estimated that 128 participants (64 per group) would be required. To account for a potential 5% attrition rate, the final target sample size was increased to 136 participants, with 68 allocated to the placebo group and 68 to the sivelestat group.

### Statistical analysis

The reporting and presentation of this trial will follow the CONSORT guideline (26). All patients will be included in the primary analysis under the intention-to-treat (ITT) principle, regardless of intervention completion, unblinding, or other protocol deviations. Supportive secondary analyses will be performed on the per-protocol (PP) population. The safety population, comprising all patients who received at least one dose of the study drug, will be used for all safety analyses.

The normality of continuous variables will be assessed using the Shapiro–Wilk test. Normally distributed continuous data will be presented as mean ± standard deviation (SD) and analyzed using the Student's *t*-test. Non-normally distributed continuous data will be presented as median with interquartile range (IQR) and analyzed using the Wilcoxon rank-sum test. Categorical variables will be presented as counts (percentages) and analyzed using the chi-square test or Fisher's exact test, as appropriate.

The primary outcome will be compared between groups using a linear regression model with treatment group and study site included as fixed effects. The estimated between-group difference with its two-sided 95% confidence interval (CI) will be derived from this model. Missing primary-outcome data within the protocol-eligible observation period will be imputed using multiple imputation by chained equations under the missing-at-random assumption, with 50 imputed datasets. The imputation model will include treatment group, study site, pre-randomization remifentanil exposure, baseline CPOT, RASS, APACHE II, SOFA, and PaO₂/FiO₂. Results will be combined using Rubin's rules and compared with a complete-case analysis. Death ends the observation period for the primary on-treatment outcome, and available data before death will be retained. No primary-outcome values after protocol-defined intervention termination, including death, will be imputed. Use of rescue analgesics will be summarized by treatment group, including the proportion of patients receiving rescue analgesics, the types of agents, and their cumulative doses. Additional opioid analgesics will be converted to morphine-equivalent doses. A sensitivity analysis will adjust for baseline PaO_2_/FiO_2_ measured before randomization. Exploratory subgroup analyses of the primary outcome will be conducted according to baseline white blood cell count and C-reactive protein level. The timing and reasons for early intervention termination will be summarized by treatment group.

For each participant, CPOT target attainment will be calculated as the proportion of valid scheduled assessments with a CPOT score ≤2. These outcomes will be analyzed using unweighted linear regression with treatment group and study site as fixed effects and HC3 heteroscedasticity-robust standard errors. The adjusted mean difference and its 95% CI will be reported in percentage points. An exploratory non-inferiority analysis will compare the lower confidence limit with −10 percentage points, which has been used in the CO-ROAM protocol (27). Supportive analyses will be performed in both the ITT and PP populations. For continuous secondary outcomes, GLMMs with Gaussian distribution and identity link will be used. Repeated continuous outcomes measured on days 3 and 7 will be analyzed using mixed models including treatment group, time, treatment-by-time interaction, baseline value, and study site as fixed effects, with participant as a random effect. Binary secondary outcomes will be analyzed using log-binomial regression adjusted for study site as a fixed effect; if convergence fails, the Mantel-Haenszel method stratified by study site will be used to estimate the relative risk (RR) and its 95% CI. If continuous outcomes violate the normality assumption, appropriate transformations (e.g., log, square root) will be considered prior to analysis.

The statistical tests of the primary outcome will be two-sided. P-values will be reported to two decimal places, and a *P*-value < 0.05 will be considered statistically significant. No interim analysis is planned for this study. There will be no adjustment for multiple comparisons. For the secondary outcomes, point estimates with 95% CI will be reported without *P* values. The widths of the CI will not be adjusted for multiplicity, so they should not be used to infer definitive treatment effects. Analyses will be performed with the use of R version 4.4.3 (R Foundation for Statistical Computing, Vienna, Austria).

A detailed Statistical Analysis Plan will be revised and finalized before database lock and unblinding. The plan will prespecify the handling of rescue analgesia, missing primary-outcome data, subgroup analyses, and alternative analytical approaches if the assumptions of the planned linear regression model are not met. The full model specification, opioid-conversion rules, and any additional exploratory analyses will also be prespecified. The exploratory CPOT analysis and the change from a random to a fixed study-site effect were introduced after Protocol Version 2.0. Their timing and rationale will be documented in the finalized Statistical Analysis Plan. Statistical analysis changes after Protocol Version 2.0 are summarized in Supplementary Table S2.

### Study status

The original protocol was Version 1.0, dated 10 January 2024. Protocol Version 2.0 was approved on 31 March 2025 (approval no. KY-2024-093-02), when 36 participants had been enrolled; the investigators remained blinded and had not reviewed treatment-specific outcome data. The amendment corrected an erroneous reference to fentanyl and refined the eligibility criteria without changing the primary outcome, sample-size calculation, remifentanil titration algorithm, rescue-analgesia protocol, or planned statistical analysis; full details are provided in Supplementary Table S1.

The trial was registered on 8 July 2024, and the first participant was enrolled on 25 July 2024. As of July 2026, 101 participants had been enrolled, representing 74.3% of the target sample size of 136. Recruitment has been limited by strict eligibility criteria, exclusion of surgical patients, and the complexity of the patient population in participating tertiary ICUs. Based on an expected recruitment rate of 4–5 participants per month, the remaining 35 participants are expected to be enrolled by March 2027.

## Discussion

To our knowledge, the SIDING trial is the first multicenter, randomized controlled trial to investigate the opioid-sparing effect of sivelestat in patients under mechanical ventilation.

The choice of remifentanil as the background analgesic in this study was primarily based on its widespread use in Chinese ICUs (27). Unlike fentanyl or sufentanil, remifentanil is metabolized by non-specific esterases independent of hepatic or renal function, with a rapid onset of analgesic action (1 min) and fast offset of action (3–10 min), allowing rapid titration and neurological assessment (28).

Importantly, the safety of sivelestat has been established in both patients with acute lung injury and healthy subjects. AEs were infrequent and generally mild, with doses up to 20.2 mg/kg/h in single-dose cohorts and 5.0 mg/kg/h in multiple-dose cohorts (29). Given the preclinical evidence, sivelestat may serve as a novel non-opioid analgesic capable of contributing to opioid-sparing multimodal regimens.

To minimize confounding factors and ensure homogeneity between groups, the eligibility criteria were designed to be stringent, excluding patients with pre-existing conditions or concurrent factors that could affect pain perception. In addition, a standardized remifentanil titration protocol and regular pain assessments were implemented to achieve analgesic targets across both groups. Mean hourly remifentanil consumption was therefore selected as the primary outcome because it provides an objective, quantitative measure of opioid sparing under a common CPOT-guided titration protocol. This measure reduces reliance on subjective clinical judgement and has been used in recent trials (30–32). Since remifentanil is reduced to the minimum effective dose in both groups, a floor effect may narrow the between-group difference and reduce the power to detect a treatment effect.

A major strength of this trial is its multicenter design, which includes seven tertiary hospitals across diverse regions of China. This enhances the external validity of the findings and ensures that the study population reflects the heterogeneity of real-world ICU practice. Furthermore, the double-blind, placebo-controlled design reduces the risk of detection and performance bias. The use of identical study drugs, prepared and dispensed by an independent pharmacy team, ensures adequate blinding of patients and clinicians. The predefined criteria for discontinuation of study drug and emergency unblinding enhance patient safety while maintaining the integrity of the trial.

This study also has some limitations. First, the study population is limited to non-surgical patients under mechanical ventilation, which may limit the generalizability of our findings to other patient populations, such as those with chronic, neuropathic, or postoperative pain. Second, the trial is conducted in Chinese ICUs, which may also limit generalizability to healthcare systems with different analgesic practices. Third, while the use of other analgesic agents is permitted per protocol, their potential effects on pain control could confound the interpretation of the primary outcome. Fourth, no pharmacokinetic interaction between sivelestat and remifentanil has been studied. Remifentanil is rapidly hydrolyzed by esterases and shares no known metabolic pathway with sivelestat, but an unexpected interaction cannot be ruled out. Fifth, this trial cannot distinguish a direct analgesic action of sivelestat from indirect effects mediated by reduced inflammation or improved pulmonary status. Therefore, any reduction in remifentanil consumption should be interpreted as a potential opioid-sparing effect rather than evidence of direct analgesic activity. Finally, remifentanil consumption is not a direct patient-centered primary outcome. The primary outcome measures remifentanil dosing intensity during the protocol-eligible intervention period, which may end because of either clinical improvement or deterioration. Therefore, it does not estimate the overall treatment effect on cumulative opioid exposure over 96 h. Future studies are required to evaluate whether reduced opioid exposure with sivelestat translates into improved patient-centered outcomes.

In conclusion, the results of this trial may reveal whether sivelestat can reduce remifentanil consumption in ICU patients under mechanical ventilation and whether it is associated with clinical improvements.
