# Supplementary material for: Efficacy and safety of sivelestat sodium hydrate adjunct to remifentanil for analgesia in patients under mechanical ventilation (SIDING trial): protocol of a multicenter, randomized, double-blinded, placebo-controlled trial
Source: Front Med (Lausanne). 2026 Sep 30;13:1907083. doi: 10.3389/fmed.2026.1907083 (PMC13663879; doi:10.3389/fmed.2026.1907083)
Supplement: Supplementary file 1 [file Table1.docx]

**Supplementary Table S1. Summary of amendments to the trial protocol**

| **Protocol item** | **Version 1.0 (10 January 2024)** | **Version 2.0 (31 March 2025)** | **Rationale and effect on trial conduct** |
| --- | --- | --- | --- |
| Background analgesic stated in the title and protocol text | Fentanyl | Remifentanil | "Fentanyl" in Version 1.0 was a clerical error. Remifentanil had been used as the standardized background analgesic from trial initiation, including for all participants enrolled before Version 2.0. Therefore, this correction did not change the intervention actually administered. |
| Age criterion | Adults aged 18-70 years | Adults aged 18 years or older | The upper age limit was removed to avoid an arbitrary restriction, broaden the eligibility of the adult ICU population, and facilitate recruitment. |
| Surgical-status criterion | Non-surgical patients | Patients who had not undergone surgery during the preceding week | The broad term "non-surgical patients" was replaced with an operationally precise time-based criterion to improve consistency in participant screening and reduce potential confounding from recent surgery-related pain. |
| Informed-consent criterion | Refusal to sign informed consent was listed as an exclusion criterion | The corresponding exclusion criterion was removed | Written informed consent was already required as an inclusion criterion; therefore, the corresponding exclusion criterion was redundant. This change did not alter participant eligibility in practice. |
| Additional analgesic requirements | No explicit exclusion criterion | Patients with analgesic requirements unrelated to mechanical ventilation, such as patients receiving radiotherapy for malignant disease, were excluded | This criterion was added to reduce potential confounding from additional sources of pain and non-protocol analgesic use. |

**Notes:** Version 2.0 was dated and approved on 31 March 2025, when 36 participants had been enrolled. The amendment was approved by the Ethics Committee of The Affiliated Huai'an No.1 People's Hospital of Nanjing Medical University (approval no. KY-2024-093-02). All 36 participants enrolled before Version 2.0 received remifentanil as the protocolized background analgesic. When the amendment was made, the investigators remained blinded to treatment allocation and had not reviewed any treatment-specific outcome data.

**Supplementary Table S2 Summary of statistical analysis change**

| **Component** | **Protocol Version 2.0** | **Revised manuscript** | **Change type** |
| --- | --- | --- | --- |
| Primary outcome model | Gaussian GLMM with identity link; treatment fixed effect and study-site random effect. | Linear regression with treatment group and study site as fixed effects. | Method change |
| Exploratory CPOT benchmark | No CPOT non-inferiority analysis or margin specified. | Added an exploratory comparison with a −10-percentage-point benchmark, with supportive ITT and PP analyses. This is not a validated confirmatory margin. | New analysis |
| CPOT analysis model | No separate model for CPOT target attainment specified. | Specified unweighted linear regression of patient-level proportions, treatment and site fixed effects, HC3 robust standard errors, and mean difference with 95% CI. | New specification |
| Rescue analgesia | Recording of additional analgesic use stated; no rescue-adjusted regression or separate exposure analysis specified. | Added separate summaries of rescue use, agent type, cumulative dose, and morphine-equivalent exposure for additional opioids. No rescue-use covariate included. | New specification² |
| PaO₂/FiO₂ sensitivity analysis | No PaO₂/FiO₂-adjusted sensitivity analysis specified. | Added sensitivity adjustment for baseline PaO₂/FiO₂ measured before randomization. | New analysis |
| Repeated secondary outcomes | Day-3 and day-7 outcomes listed; Gaussian GLMMs stated without a longitudinal model specification. | Specified treatment, time, treatment-by-time interaction, baseline value, and site as fixed effects; participant as a random effect. | Further specification |
| Binary secondary outcomes | Log-binomial regression, with site-stratified Mantel–Haenszel estimation if convergence failed. | Explicitly specified site as a fixed effect in log-binomial regression. Existing Mantel–Haenszel fallback retained. | Further specification |
| Subgroup analyses | Primary-outcome analyses in prespecified subgroups mentioned; subgroup variables not named. | Named baseline WBC and CRP and designated subgroup analyses as exploratory. | Further specification |

All listed revisions were made on 27 August 2026 while treatment allocation remained blinded, with 104 participants enrolled.

APACHE II, Acute Physiology and Chronic Health Evaluation II; CI, confidence interval; CPOT, Critical-Care Pain Observation Tool; CRP, C-reactive protein; GLMM, generalized linear mixed model; ITT, intention-to-treat; MAR, missing at random; MICE, multiple imputation by chained equations; PP, per-protocol; RASS, Richmond Agitation-Sedation Scale; SAP, statistical analysis plan; SD, standard deviation; WBC, white blood cell.
